# Time-of-day changes in permethrin susceptibility and metabolic gene expression in Florida *Aedes aegypti* (Diptera: Culicidae)

**DOI:** 10.1093/jme/tjaf013

**Published:** 2025-02-03

**Authors:** Sierra M Schluep, Tse-Yu Chen, Shelley A Whitehead, Eva A Buckner

**Affiliations:** Florida Medical Entomology Laboratory, Department of Entomology and Nematology, Institute of Food and Agricultural Sciences, University of Florida, Vero Beach, FL, USA; Florida Medical Entomology Laboratory, Department of Entomology and Nematology, Institute of Food and Agricultural Sciences, University of Florida, Vero Beach, FL, USA; Department of Entomology, Center for Infectious Disease Dynamics, Pennsylvania State University, University Park, PA, USA; Florida Medical Entomology Laboratory, Department of Entomology and Nematology, Institute of Food and Agricultural Sciences, University of Florida, Vero Beach, FL, USA; Florida Medical Entomology Laboratory, Department of Entomology and Nematology, Institute of Food and Agricultural Sciences, University of Florida, Vero Beach, FL, USA

**Keywords:** *Aedes aegypti*, permethrin, circadian rhythm, metabolic resistance, gene expression

## Abstract

*Aedes aegypti* (Linnaeus) is the principal mosquito vector for many of the most medically significant arboviruses that threaten global public health. A better understanding of time-of-day variation in insecticide resistance mediated by detoxifying enzymes in *Ae. aegypti* could allow for targeted insecticide applications when susceptibility is highest and the upregulation of detoxification enzymes is lowest. Using a susceptible and metabolically resistant field *Ae. aegypti* strain from Florida, we investigated simulated photoperiodic changes in permethrin susceptibility and upregulation of detoxification enzymes by measuring permethrin LD_50_ and expression of detoxification genes (*GSTE7*, *GSTE2*, *CCEae3A*, *CYP9J28*, and *CYPBB2*) for both strains every 4 h over a 24 h (12:12 h light: dark) cycle. We found that in both *Ae. aegypti* strains, permethrin susceptibility was lower during the day as compared to evening, with susceptibility lowest at dusk (18:00) and highest between 02:00 and 14:00. Although no significant changes in gene expression over time were observed in the susceptible *Ae. aegypti* strain, we documented increased expression of all investigated detoxification genes in the metabolically resistant field *Ae. aegypti* strain during the night (18:00 to 02:00) as compared to the day (06:00 to 14:00). These data suggest that permethrin applications made between midnight and dawn (06:00) may be more effective against *Ae. aegypti* as compared to applications made at dusk (approximately 18:00).

## Introduction

Insects exhibit circadian rhythms that elicit behavioral and physiological responses. These rhythms are controlled by a set of highly conserved, endogenous clock genes (Hall 2003). The circadian clock is comprised of a series of transcriptional-translational feedback loops that can persist through experimental conditions of constant darkness but are often entrained by an external stimulus such as light or temperature (Clements 1999, Giebultowicz 2001, Stanewsky 2003, Schibler 2007, and Tobback et al. 2011). In insects, photoperiodicity contributes to the timing of diapause, growth rate, metabolism, and behavior (Sharma 2003). In mosquitoes, many biological and behavioral aspects (ie sugar and blood feeding, mating, oviposition, flight, odorant sensitivity, hormone release, and metabolism) are influenced by the circadian clock’s coordination with rhythmic environmental cues (Clements 1999, Hardin 2006, Rund et al. 2013a, Balmert et al. 2014, Leming et al. 2014, Duffield 2024). Rhythmic susceptibility to insecticides has been reported in numerous insects including mosquitoes (Beck 1963, Cole and Adkisson 1964, Bainbridge et al. 1982, Onyeocha and Fuzeau-Braesch 1991, Hooven et al. 2009, Yang et al. 2010, Ptitsyn et al. 2011, Rund et al. 2011, Hamby et al. 2013, Balmert et al. 2014, Piechowicz et al. 2016, Khalid et al. 2019, Odufuwa et al. 2024).

Insecticide resistance has been shown to be influenced by photoperiodic entrainment of the circadian clock in mosquitoes. For example, Yang et al. (2010) documented increased resistance as measured by increased mean knockdown time in both susceptible and permethrin-resistant *Aedes aegypti* (Linnaeus) strains during the light phase when entrained by 12:12 h L:D cycle and constant darkness (0: 24 h), with overall levels of resistance being higher in the photoperiodic cycle than the circadian rhythm alone. Likewise, Villanueva et al. (2016) detected increased permethrin resistance, measured by increased LC_50_ values, in multiple *Ae. aegypti* populations from southern Mexico and a susceptible *Ae. aegypti* reference strain when entrained by a 12:12 h L:D cycle as opposed to constant darkness.

Furthermore, the expression of specific genes in mosquitoes responsible for controlling the regulation of detoxification enzymes has also been shown to be influenced by photoperiodic entrainment of the circadian clock (Yang et al. 2010, Ptitsyn et al. 2011, Rund et al. 2013b, Balmert et al. 2014, Leming et al. 2014). Yang et al. (2010) demonstrated that the expression levels of the detoxification enzyme *CYP9M9* were higher when entrained by a light: dark cycle than by constant darkness in both susceptible and resistant *Ae. aegypti*, with overall significantly higher expression observed in the permethrin-resistant strain (peaking at ~17:00 followed by a trough at ~21:00). Similarly, Rund et al. (2013b) found that there are a large proportion of *Anopheles gambiae* Giles genes, including those involved in metabolic detoxification like *GSTE5* and *CYP6M2*, that have expression driven by the environmental light: dark cycle as opposed to constant darkness.

Studying the chronotoxicity of *Ae. aegypti* is of particular importance considering its status as the worldwide principal vector of chikungunya, dengue, yellow fever, and Zika viruses. Increased international travel, global climate change, and urban growth contribute to the seamless expansion of *Ae. aegypti* and its suitable habitats (Ryan et al. 2019, Satoto et al. 2019). Its presence on every continent, excluding Antarctica, equates to over half of the world’s population being at risk of contracting an *Aedes-*borne virus (Kraemer et al. 2016). In recent years, outbreaks of locally transmitted *Aedes-*borne diseases have occurred in Florida, resulting in 11 cases of chikungunya virus in 2014 and approximately 300 cases of Zika virus in 2016 (FDOH 2014, Likos et al. 2016), Additionally, sporadic dengue cases or outbreaks have occurred every year in Florida since 2010, with a recent dengue outbreak in 2023 resulting in 176 documented cases of locally acquired dengue (FDOH 2023).

While a successful integrated mosquito management plan used to control *Ae. aegypti* should include multiple interventions such as larval mosquito habitat elimination through source reduction, biological control, and community engagement, it will also most likely contain insecticide treatment of larvae and/or adults. In the United States, pyrethroids are one of the 2 classes of insecticides used against adult mosquitoes. Specifically in the state of Florida, permethrin is the most used pyrethroid insecticide for controlling adult mosquitoes (Lloyd et al. 2018), and it is speculated that over-reliance on permethrin has led to the presence of resistance in populations of *Ae. aegypti* throughout the state (Estep et al. 2018, Parker et al. 2020, Schluep and Buckner 2021, Scott et al. 2021). Understanding if and how time-of-day influences permethrin susceptibility and metabolic gene expression in Florida *Ae. aegypti* could potentially lead to increased efficacy of adulticidal sprays due to the scheduling of applications at the time-of-day when mosquitoes are most susceptible. Therefore, we investigated if permethrin susceptibility varies throughout the photoperiod in a field *Ae. aegypti* strain collected from Florida that we previously found to be metabolically resistant to permethrin (Schluep and Buckner 2021). Additionally, we investigated the relationship between any potential time-of-day variation observed in permethrin susceptibility and variation in the expression levels of 5 metabolically significant genes (*CYP9J28, CYP6BB2, GSTE7, GSTE2,* and *CCEae3A*) (Strode et al. 2008, Poupardin et al. 2014, Marcombe et al. 2019, Rahman et al. 2021). To our knowledge, this is the first study to investigate the correlation between photoperiodic changes in permethrin susceptibility and metabolic gene expression in field *Ae. aegypti* mosquitoes from Florida and the United States.

## Materials and Methods

### Mosquito Rearing

The Cheyenne (CHY) *Ae. aegypti* field strain used in experiments was collected from Polk County, Florida (28.14449, −82.00326) in August 2020 and exhibits metabolic resistance to permethrin (Schluep and Buckner 2021). To obtain the CHY *Ae. aegypti,* eggs were collected on seed germination paper placed inside black oviposition cups left in the field for 5 to 7 d. Eggs of both ORL and CHY *Ae. aegypti* strains were hatched in the laboratory and reared to larvae in 40.6 × 15.4 × 6.4 cm enamel pans containing approximately 2 L of tap water at a density of 250 larvae per pan. Pupae originating from field-collected eggs were transferred from larval-rearing trays to water-filled cups in 30.5 × 30.5 × 30.5 cm Bug Dorm adult rearing cages (Bioquip Products, Rancho Dominquez, CA). A cotton ball soaked with 10% sucrose solution was placed inside each cage as a carbohydrate source for P_1_ adults that emerged. Adults of at least 3 d old were provided a bloodmeal twice a week from a live chicken (UF IACUC Protocol # 201807682) and allowed to feed for 45 min. This process was repeated for F_1_ adults for the collection of the required number of F_2_ eggs. All experiments were performed with the F_2_ generation of the permethrin-resistant field strain (CHY). All life stages of the mosquitoes were kept in a walk-in insectary maintained at 27 °C ± 2 °C and 70% ± 5% relative humidity with a 12:12 h L:D photoperiod including a 1 h dusk (17:30 to 18:30) period and 1 h dawn (05:30 to 06:30) period. The 1 h dawn and dusk period were simulated by having a small desk lamp turn on at hours 05:30 and 17:30 until the insectary-wide overhead lighting turned on at 6:30 and 18:30. The Orlando 1952 (ORL) *Ae. aegypti* strain was used as a susceptible reference strain in this study and reared under the same conditions described above for the CHY *Ae. aegypti* strain. The ORL strain is originally from Orlando, FL and has been maintained in the laboratory since 1952.

### Topical Chronotoxicity Assays

Technical grade permethrin (100%, Chem Service Inc., West Chester, PA) was used for the topical applications. The adult mosquito topical bioassay has been described previously in detail (Pridgeon et al. 2008, Estep et al. 2018). An initial 1 mg/ml stock solution of permethrin diluted with acetone (Thermo Fisher Scientific, Waltham, MA) was prepared to create a series of sub-dilutions necessary to induce 0% to 100% mortality, targeting the critical region of the dose curve, for both the ORL and CHY strains. Five permethrin-acetone dilutions were used for both strains (Table 1). Both strains also received a controlled dose of pure acetone for each set of replicates, and 4 replicates at each time-point were performed for both control and 5 permethrin-acetone dilutions.

**Table 1.** Doses in parts per million (PPM) of permethrin-acetone dilutions that induced 0% to 100% mortality in topical chronotoxicity assays against the permethrin-resistant field *Aedes aegypti* strain (CHY) and susceptible *Ae. aegypti* lab strain (ORL).

CHY		ORL	
Dose (mg/ml)	Dose (PPM)	Dose (mg/ml)	Dose (PPM)
0.00	0	0.0000	0.0
0.01	10	0.0010	1.0
0.10	100	0.0025	2.5
0.25	125	0.0050	5.0
0.50	500	0.0100	10.0
1.00	1,000	0.0500	50.0

Before topical application of permethrin-acetone dilutions, 4- to 7-d-old adult female mosquitoes were briefly anesthetized for 1 min 45 s in a −20 °C freezer and placed on a −4 °C laboratory chill table (BioQuip Products, Ranch Dominquez, CA). A volume of 0.1 μl of permethrin solution was applied acutely to the dorsal thorax of each adult female using a 700 series 5 μl volume syringe (Hamilton, Reno, NV) and a PB 600 repeating dispenser (Hamilton, Reno, NV). Four replicates of 25 to 30 females each were treated with the 6 doses performed at 6 zeitgeber time points throughout the 12:12 h L:D cycle, 06:00, 10:00, 14:00, 18:00, 22:00, and 02:00. For treatments performed in the dark phase, a red light was used to handle mosquitoes. After treatment, mosquitoes were kept in 8-oz poly-coated paper cups (WebstaurantStore, Lancaster, PA) with lids replaced with tulle (holding cups) and provisioned with a cotton ball soaked in 10% sucrose solution. The mosquitoes in the holding cups were left in the walk-in insectary overnight and a 24-h reading of mortality was recorded before freezing and counting the mosquitoes.

The LD_50_ was calculated for each timepoint for both the CHY and ORL strains using the probit regression function in IMB SPSS statistical software platform. Corresponding 95% confidence intervals (C.I.), parameter estimates (slope ± standard error), and the Chi-Squared goodness of fit statistic (measures how well the observed distribution of data fits within the distribution that is expected if the variables are independent) were also outputs of the probit regression. Results for the 2 strains were considered to have significantly different LD_50_ values if the 95% confidence intervals did not overlap. Control mortality did not need correction using Abbott’s formula as all control mortality was < 4.1% (Abbott 1925).

### Comparison of Detoxification Gene Expression Levels Using qPCR

Adult female *Ae. aegypti* from both the CHY and ORL strains were collected every 4 h across a 24-h period at 06:00, 10:00, 14:00, 18:00, 22:00, and 02:00 by briefly being anesthetized for 1 min 45 s in a −20 °C freezer. Mosquitoes were transferred in groups of 5 into RNase free 1.5 ml microcentrifuge tubes and immediately placed into a −80 °C freezer. For each strain, 4 replicate samples were taken at all 6 timepoints. Gene-specific primers were obtained for one reference gene, *RpS7* (Chen et al. 2021) and 5 target genes, *GSTE7* (Lima et al. 2015)*, GSTE2* (Chen et al. 2021) *CCEae3A* (Marcombe et al. 2019)*, CYP9J28* (Marcombe et al. 2019), and *CYPBB2* (Marcombe et al. 2019). Melt curve and BLAST analysis were used as criteria to confirm primer specificity. The gene expression level was assessed at the 6 timepoints for both the ORL and CHY strains.

Total RNA was extracted from whole body lysates in pools of five 4- to 7-d-old female mosquitoes using TRIzol reagent following the manufacturer’s protocol (Sigma-Aldrich, St. Louis, MO). Mosquitoes were placed in 1.5 ml tubes containing 1 ml of TRIzol and eight 2 mm glass beads (Sigma-Aldrich). Samples were then homogenized using a Bullet Blender Storm (Next Advance Inc, Troy, NY). Total RNA quality and quantity were checked using a Nanodrop 2000 spectrophotometer (Thermo Fisher Scientific).

Genomic DNA was removed by digesting total RNA samples with DNase I (RNase-free) (Invitrogen Ambion, Thermo Fisher Scientific). 10× DNase I Buffer was added to 1× concentration in the solution to be DNase-treated, and approximately 1 to 2 U of DNase I per 1 μg DNA present was added to create a 20-μl volume of DNase-treated RNA. DNase I was inactivated by 10 min incubation at 75 °C. Total RNA quantity and quality following DNase treatment was assessed by spectrophotometry using the Nanodrop 2000.

cDNA synthesis was performed using SuperScript IV First Strand Synthesis System (Invitrogen, Thermo Fisher Scientific) following the standard protocol. Quantitative real-time PCR (qPCR) was performed in duplicates using the PowerUPSYBR Green Master Mix (AppliedBiosystems, Thermo Fisher Scientific) consisting of 100 ng cDNA in a total reaction volume of 20 μl. Components of the reaction consisted of 10 μl Master Mix, 1 μl forward primer, 1 μl reverse primer, 3 μl of RNase-free water, and 5 μl of 20 ng cDNA. Prior to use in qPCR, primers were diluted to 10 µM to generate working stocks. qPCR conditions included one cycle of 50 °C for 2 min, one cycle of 95 °C for 2 min, followed by 40 cycles of 95 °C for 15 s and 60 °C for 1 min using the CFX96 Touch Real-Time PCR Detection System (Bio-Rad Laboratories, Inc., Hercules, CA). Before data analysis, quantification cycle (cq) data were checked, and sample duplicates with cq standard errors of greater than 1.0 or that failed to produce a cq value were re-prepared from extracted RNA for qPCR. Data analysis was performed according to the ΔΔCt value method using the *RpS7* gene to normalize expression levels (Livak and Schmittgen 2001).

### Expression Consistency of Reference Gene

Because this portion of the study aimed to examine gene expression changes across timepoints, the reference gene *RpS7* (AAEL009496) was checked for integrity across timepoints. A Tukey test for multiple comparisons was performed in R version 4.0.4 (RStudio Inc., Boston, MA) that created a set of confidence intervals on the differences between the means of both reference genes individually (relative fold change relative to 06:00 h) and was fitted to ANOVA. Only *P* < 0.05 was counted as significantly different.

### ORL vs CHY Average Fold Change at Each Timepoint

To compare the average fold change for gene expression between the CHY and ORL strains at each of the 6 timepoints, the non-parametric Wilcoxon rank-sum test (Mann–Whitney *U* test) statistic was performed in R version 4.0.4 (RStudio Inc., Boston, MA). Only *P* < 0.057 were considered significantly different. Significant *P*-value outputs were either 0.029 (*GSTE2* and *CCEAE3A*) or 0.057 (*CYP6BB2, CYP9J28*, *GSTE7*). Typically, 0.05 is the standard *P*-value for assigning significance; however, an additional 0.007 is an artifact of the strength and type of analysis used.

### Differences in Gene Expression Across Time for Each Strain

To investigate if there were differences in gene expression at each timepoint for the CHY strain and ORL strain independently, the fold difference values for each timepoint were compared using the *RpS7* reference gene. A Kruskal–Wallis (KW) non-parametric test was performed in R version 4.0.4 (RStudio Inc., Boston, MA) to test whether the mean ranks (relative fold change) were the same in all the groups. A post-hoc Dunn’s multiple comparison test was run after the KW with the Bonferroni *P*-value adjustment. Only *P* < 0.05 was counted as significantly different. For each comparison, results are expressed as mean transcription ratios (± SE) (Livak and Schmittgen 2001).

## Results

### Topical Chronotoxicity Assays

**Fig. 1.**
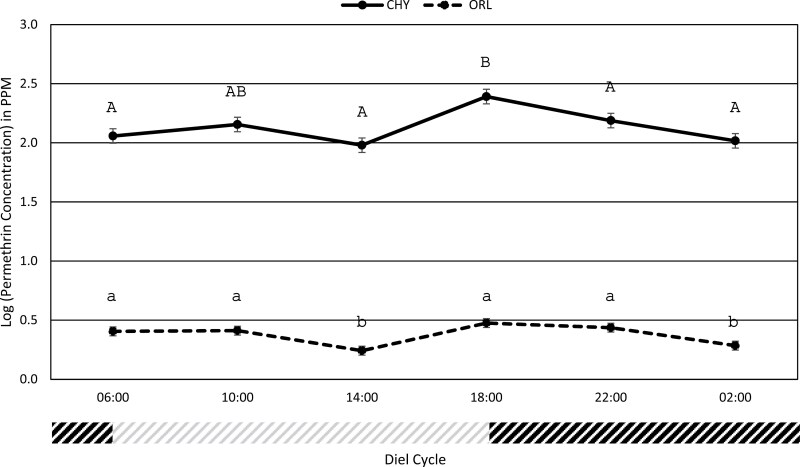
The permethrin LD50 of the resistant field *Aedes aegypti* strain (CHY) and the susceptible Aedes aegypti lab strain (ORL) expressed in log concentration PPM at 6 different timepoints throughout the 24-h diel cycle. Different uppercase (CHY) and lowercase (ORL) letters indicate significant differences (95% C.I. do not overlap) between timepoints. A timeline representing the experimental 12 h light:12 h dark cycle during the selected timepoints is provided. The gray portion of the timeline indicates light phase and black portion indicates dark phase.

An initial broad-range topical assay for the permethrin-resistant *Ae. aegypti* strain (CHY) and the susceptible reference *Ae. aegypti* lab strain (ORL) demonstrated that the doses of permethrin solution required to target the critical region of the dose curve and accurately calculate the LD_50_ were 10 to 50-fold higher for the CHY *Ae. aegypti* strain compared to the doses required for the ORL *Ae. aegypti* strain (Table 1). Concurrently, mean LD_50s_ at different time points were 55 to 141 times higher for the CHY strain compared to the ORL strain (Tables 2 and 3). Regardless of differences in permethrin susceptibility, the LD_50_ over time for both strains followed a similar trend with the lowest LD_50_ documented at the 14:00 and 02:00 timepoints and the highest LD_50_ documented at the 18:00 timepoint (Fig. 1).

**Table 2.** The permethrin LD_50_ (PPM) and Resistance Ratio (RR) of the resistant field *Aedes aegypti* strain (CHY) at 6 different timepoints throughout the 12 h light:12 h dark cycle.

Timepoint (24 h)	N	LD_50_ (95% C.I.)	Slope ± SE	χ^2^	RR
06:00	604	114.23 (93.68 to 135.39)	2.17 ± 0.18	1.20	44.80
10:00	601	142.99 (56.17 to 268.26)	1.71 ± 0.14	12.97	55.42
14:00	606	95.57 (45.03 to 147.06)	2.60 ± 0.24	9.19	54.61
18:00	590	246.37 (187.53 to 315.17)	3.47 ± 0.29	5.68	82.40
22:00	599	154.33 (127.20 to 182.79)	1.95 ± 0.17	1.10	56.32
02:00	601	104.06 (51.88 to 166.16)	2.07 ± 0.17	8.77	53.92

**Table 3.** The permethrin LD_50_ (PPM) of the susceptible *Aedes aegypti* lab strain (ORL) at 6 different timepoints throughout the 12:12 h cycle.

Timepoint (24 h)	N	LD_50_ (95% C.I.)	Slope ± SE	χ^2^
06:00	583	2.55 (2.28 to 2.83)	3.63 ± 0.30	0.37
10:00	597	2.58 (2.32 to 2.86)	3.64 ± 0.29	2.73
14:00	597	1.75 (1.26 to 2.27)	3.60 ± 0.32	6.43
18:00	592	2.99 (2.67 to 3.34)	3.22 ± 0.26	3.59
22:00	601	2.74 (2.47 to 3.01)	3.97 ± 0.33	0.14
02:00	615	1.93 (1.73 to 2.13)	3.99 ± 0.34	3.85

The LD_50_ for the permethrin-resistant CHY *Ae. aegypti* strain at 06:00, 10:00, 14:00, 22:00, and 02:00 did not significantly differ, ranging from 95.57 PPM to 154.33 PPM, while the LD_50_ at 18:00 was significantly higher with a LD_50_ of 246.37 PPM (Fig. 1, Table 2). The LD_50_ for the ORL *Ae. aegypti* strain at 06:00, 10:00, 12:00 and 22:00 did not significantly differ, ranging from 2.55 PPM to 2.99 PPM. The timepoints with the lowest LD_50_ for the ORL *Ae. aegypti* strain were 14:00 (1.75 PPM) and 02:00 (1.93 PPM), which were significantly lower than the other timepoints (Fig. 1, Table 3).

The resistance ratio (RR) for the CHY strain at the timepoints measured ranged from 44.80 to 82.40, indicating that the population is highly resistant to permethrin compared to susceptible the ORL strain (RR = 1) (Table 2) (Chouaibou et al. 2012, Estep et al. 2018). The highest RR (82.40) in the CHY strain was documented at the timepoint 18:00, which had a significantly higher LD_50_ compared to all other timepoints, suggesting that the CHY strain was at peak resistance to permethrin during this time of day.

### Expression Consistency of Reference Genes

**Fig. 2.**
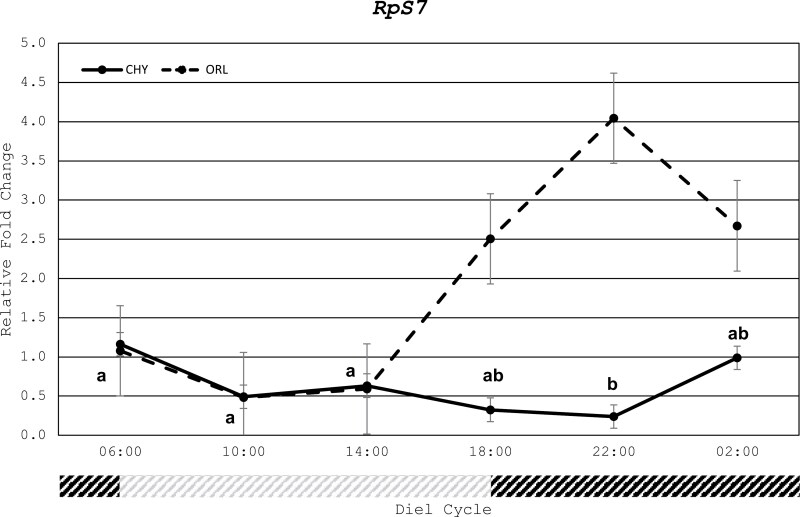
Differences in relative fold change from 06:00 for the reference gene RpS7 (AAEL009496) across all 6 different timepoints throughout the 12:12h cycle in resistant field Aedes aegypti strain (CHY) and susceptible *Aedes aegypti* lab strain (ORL). Different lowercase letters indicate significant differences in the CHY strain. The gray portion of the timeline indicates light phase and black portion indicates dark phase.

One reference gene, *RpS7*(AAEL009496) was selected for expression level normalization. The *RpS7* primer obtained from Chen et al. (2021) was stable across all timepoints for the CHY strain with a range of 0.24 to 1.16 for the CHY strain and 0.48 to 4.04 for the ORL strain (Fig. 2). The stability across 24 h seen here is consistent with the profile of this gene in *Ae. aegypti* from the bioclock database (Leming et al. 2014). For the subsequent analysis of gene expression between the 2 strains and across timepoints for each strain individually the cq values normalized to *RpS7* were used.

### Average Fold Change at Each Timepoint—ORL vs CHY Strain

**Fig. 3.**
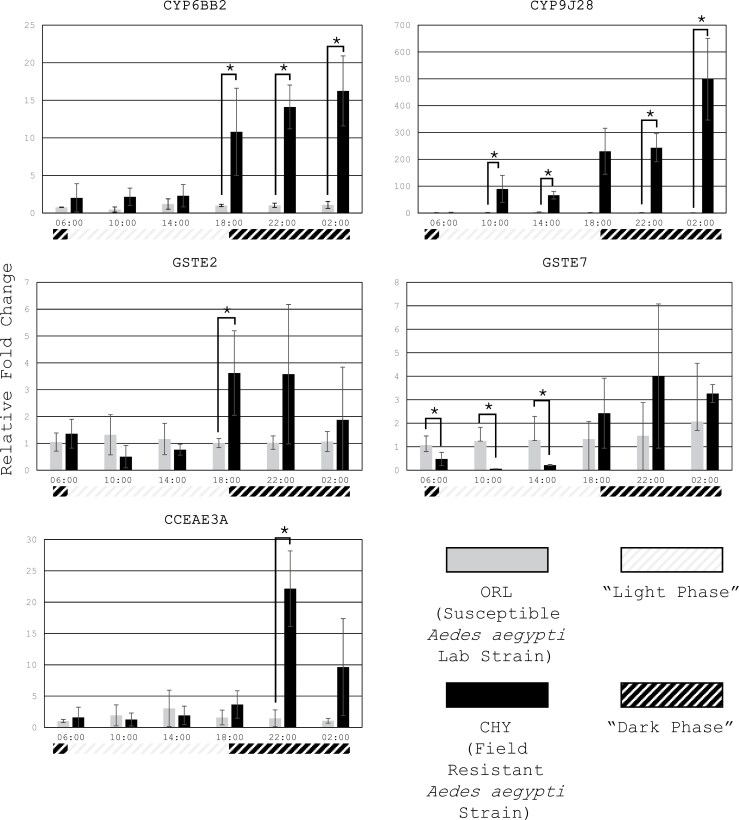
Average fold change between the *Ae. aegypti* permethrin-resistant field strain (CHY) and susceptible lab strain (ORL) for each gene individually at each of the 6 timepoints using RpS7 as the reference gene. The gray portion of the timeline indicates light phase and black portion indicates dark phase. * indicates significant differences (*P* ≤ 0.057 or *P* = 0.029).

By comparing the mean relative fold change in gene expression for 5 metabolic detoxification genes at 6 timepoints between the ORL and CHY strains, we found that the most significant differences in expression were in the CHY strain and the highest expression levels of these genes were in the dark phase. Contrastingly, the ORL strain exhibited gene expression that remained consistent over time (Fig. 3).

In the CHY strain, significantly higher expression levels were exhibited compared to ORL in *CYP6BB2* at 18:00 (*P* = 0.029), 22:00 and 02:00 (*P* ≤ 0.057); *CYP9J28* at 10:00, 14:00, 22:00, and 02:00 (*P* ≤ 0.057), *GSTE2* at 18:00 (*P* = 0.029), and *CCEae3A* at 22:00 (*P* = 0.029). Although *GSTE7* expression levels were not significantly different in CHY from ORL at 18:00, 22:00, and 02:00 due to high standard deviations, ORL expression of *GSTE2* was significantly higher than CHY at 06:00, 10:00, and 14:00 (*P* ≤ 0.057).

### Differences in Gene Expression Across Time for Each Strain

**Fig. 4.**
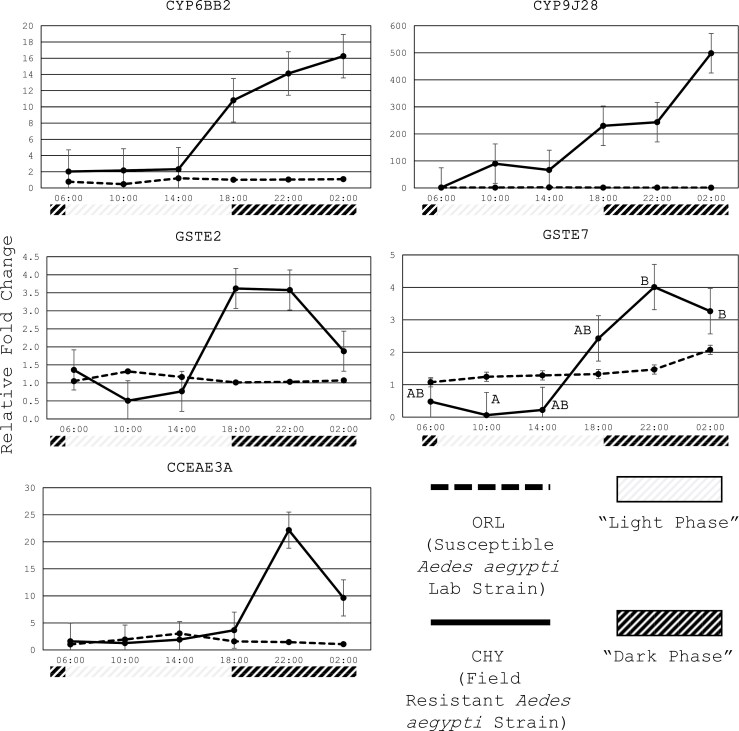
Average fold change across timepoints for the CHY and ORL populations individually using RpS7 as the reference gene. Different letters indicate significant differences (*P* ≤ 0.05). The gray portion of the timeline indicates light phase and black portion indicates dark phase.

By comparing the mean relative fold change between each timepoint for the CHY strain and ORL strain individually, we revealed temporal expression patterns only in the CHY strain. In that strain, the genes exhibited a general trend of increased expression in the dark phase (18:00 to 02:00). While the change in gene expression was only significantly different (*P* ≤ 0.05) between 10:00 and 22:00 to 02:00 for *GSTE7*, expression for all 5 genes followed a similar trend, which was expression starting to increase after 14:00, with expression reaching its highest in the dark phase between 18:00 and 02:00. The lower expression levels during 06:00-14:00 coincide with the light phase. None of the genes demonstrated statistical differences in expression levels throughout the 12:12 h L:D cycle for the ORL strain, which remained stable throughout the 12:12 h cycle (Fig. 4).

## Discussion

To our knowledge, this is the first time that photoperiodic variation in insecticide susceptibility has been studied in an *Ae. aegypti* field strain from the continental U.S. The aim of this study was to evaluate chronotoxicity to permethrin and the potential metabolic resistance mechanism using a resistant field strain of *Ae. aegypti* (CHY) entrained by a 12:12 h L:D cycle. We found that both the susceptible lab (ORL) and resistant field strain (CHY) of *Ae. aegypti* mosquitoes exhibited variations in permethrin susceptibility over the 24-h diel cycle, with strains requiring the highest LD_50_ at 18:00 and the lowest LD_50_ at 14:00. In the CHY strain, peak in resistance was followed by a phase of increasing susceptibility, which began at 22:00 and continued into the early morning (02:00). Regarding the expression of selected metabolic enzymes, expression levels remained stable throughout the 12:12 h cycle in the ORL strain while the CHY strain exhibited a general trend of increased expression after 14:00, with expression reaching its highest in the dark phase between 18:00 and 02:00.

It is well documented that external stimuli, such as light, can influence behavioral and physiological aspects of mosquitoes including flight activity, oviposition, host-seeking, and blood-feeding (Taylor and Jones 1969, Chadee 1988, Clements 1999, Rund et al. 2020). The phase of highest resistance is speculated to be correlated with the daily patterns of activity in mosquitoes (Yang et al. 2010, Odufuwa et al. 2024). For example, Odufuwa et al. (2024) evaluated variations in susceptibility throughout the 24-h day on metabolically pyrethroid-resistant *Anopheles arabiensis* Patton and *Culex quinquefasciatus* Say finding increased resistance in both species during the night (16:00 to 24:00), when these nocturnal mosquitoes are most active and likely exhibit higher metabolic activity, compared to the day (09:00 to 14:00) (Balmert et al. 2014, Odufuwa et al. 2024). *Aedes aegypti* has primarily been reported as a daytime active species, with small peaks of activity at dawn, increased activity during the afternoon, and larger peaks in activity just prior to dusk (~12:00 to 18:00) (Taylor and Jones 1969, Corbet and Smith 1974, Jones 1981, Yee and Foster 1992, Chadee 2010, Wong et al. 2011). The peak of permethrin resistance at dusk (18:00) that we documented in both *Ae. aegypti* strains correlates with a peak in *Ae. aegypti* activity. We also observed climbing resistance to permethrin from 14:00 to 18:00 in our resistant strain, which also overlaps with known *Ae. aegypti* diurnal activity. However, our findings differed from those of Yang et al. (2010) with their artificially selected permethrin-resistant strain of *Ae. aegypti* exhibiting higher levels of resistance at ~07:00.

There are many enzymes involved in the metabolic detoxification of insecticides that are found to be expressed in rhythms and may contribute to increased resistance during certain times of day (Strode et al. 2008, Yang et al. 2010, Ptitsyn et al. 2011, Rund et al. 2013b, Balmert et al. 2014, Leming et al. 2014). Preliminary data revealed a significant impact of esterase, cytochrome p450, and glutathione-s-transferase inhibitors on the susceptibility to permethrin of the resistant *Aedes aegypti* strain (CHY), indicating the presence of metabolic resistance (Schluep and Buckner 2021). We hypothesized that the time-phase of peak resistance (dark phase; 18:00) in this metabolically resistant population would correlate with the time-phase of peak expression of these genes encoding pyrethroid metabolizing and sequestering enzymes (Hooven et al. 2009, Hamby et al. 2013, Villanueva et al. 2016); All of which were previously found to be under light regulation in the Higgs white eye *Ae. aegypti* strain (Leming et al. 2014).

In this experiment, the resistant strain (CHY) exhibited increases in the expression of all selected genes in the dark phase (18:00 to 02:00) when compared with the susceptible strain (ORL). These samples were taken from untreated mosquitoes; therefore, there was no induction or sublethal exposure to permethrin and the results herein are what we predict are constitutively overexpressed patterns due to the selective environmental pressures in this resistant population (Strode et al. 2008). From 06:00 to 14:00, gene expression values of the resistant strain (CHY) were more similar to and/or overlapping with those of the susceptible strain (ORL), which remained consistent across all timepoints. Although all genes were more highly expressed during the dark phase, no defined peak in expression was demonstrated at 18:00 in correlation with our time of peak resistance other than *GSTE2* (a known DDT metabolizer), which was most highly expressed at 18:00 and 22:00. It is worth noting that while our study focused on measuring mRNA, peaks in mRNA levels may not necessarily correspond with peak protein as demonstrated by Rund et al. (2013a) finding that *GSTE3* of *Anopheles gambiae* exhibited rhythmic mRNA levels but observed corresponding constitutive protein levels (Rund et al. 2013a). The comparisons between gene expression (ie mRNA level) made herein are amongst studies investigating similar enzymes implicated in insecticide detoxification in *Ae. aegypti*.

Our findings are similar to those of other studies. For example, in Yang et al. (2010), C*YP9M9* was proven to have an influence on pyrethroid resistance in *Ae. aegypti* as well as be regulated by *per* (a circadian clock regulating gene) as *per* silencing led to lower expression of *CYP9M9* and reduced resistance to permethrin. However, peak enzyme activity (~17:00) did not correlate with the phase of highest resistance in the permethrin-resistant *Ae. aegypti* strain (~07:00) (Yang et al. 2010). Similarly, Balmert et al. (2014) found peak expression of 3 *CYP* genes (*CYP693*, a pyrethroid metabolizer; *CYP6M2*, a type I and II pyrethroid metabolizer; and *CYP6Z1*, a DDT metabolizer) at ~22:00 to 23:00 in their susceptible *An. gambiae* strain. However, this strain exhibited a peak in DDT resistance at ~17:00 in and deltamethrin resistance at ~07:00 and ~17:00 (Balmert et al. 2014). Interestingly, *GSTE2* (DDT metabolizer) expression peaked at ~08:00, not in line with peak DDT resistance, but more synchronized with the first peak in deltamethrin (type II pyrethroid) resistance at ~07:00.

Florida mosquito control programs most often apply permethrin in the form of ultra-low volume (ULV) space sprays to kill flying adult mosquitoes when they are active (Lloyd et al. 2018). Thus, local mosquito control programs conduct ULV sprays targeting *Aedes* mosquitoes at dawn or dusk to account for flight activity as well as to mitigate the reduced photostability of products and the effects of daytime high temperatures on the efficacy of product application (Lloyd et al. 2018). The mosquito control program, Polk County Mosquito Control, where the resistant CHY *Ae. aegypti* field strain used in this study originates, has historically conducted their adulticide treatments with pyrethroids around dusk (~18:00). We observed that CHY *Ae. aegypti* exhibited its highest peak in permethrin resistance at dusk, the onset of the dark phase (18:00), and that the genes that encode for the enzymes known to play a role in pyrethroid resistance were all expressed at higher levels during the dark phase (18:00 to 02:00). These observations may suggest that the historical practice of dusk-time permethrin application targeting adult mosquitoes in this area might have contributed to increased permethrin resistance in *Ae. aegypti* during this time of day and should be further explored in various field populations.

The potential relationship between active ingredient, application time, and mosquito susceptibility (ie chronotoxicity) may provide a valuable pathway for future insecticide resistance evaluation if more field populations show tolerance to permethrin and/or corresponding upregulation of detoxification enzymes at a particular time of day. Based on our limited findings, we suggest that it may be worthwhile for mosquito control programs that have historically made ULV permethrin space spray applications at dusk to consider shifting application times from dusk to near the end of the dark phase (ie after 02:00) when targeting *Ae. aegypti*. Applications made at this time of day, when *Ae. aegypti* is most susceptible to permethrin and metabolic enzyme activity is lowest, which may allow mosquito control programs to be able to apply a lower rate of insecticide, improving operational cost-effectiveness (Hamby et al. 2013). Furthermore, pre-dawn ULV permethrin space spray applications would continue reduce potential non-target impacts since pollinators are generally more active during daytime hours (Willmer 1983, Lehmann et al. 2011, Knop et al. 2018, Karbassioon and Stanley 2023). Also, targeting *Ae. aegypti* around peak susceptibility timepoints could be a way to curb the propagation of metabolic resistance by preventing sublethal exposure, which is currently seen in many *Ae. aegypti* populations throughout Florida (Schluep and Buckner 2021).

It is important to note that we tested only 5 metabolically significant genes in one field permethrin-resistant strain of *Ae. aegypti* and one susceptible reference strain of *Ae. aegypti*. In addition to the activity levels and expression of metabolic detoxification or sequestering genes, there are likely other factors that might contribute to the chronotoxicity seen here, such as antioxidant levels (Beaver et al. 2012). It would be beneficial to determine if the chronotoxicity found for the resistant CHY field strain is similar to other field strains of *Ae. aegypti* both from Florida and other regions in United States to further confirm a correlation between the timing of spray operations and peaks in resistance.
